# Regeneration of Stereocilia of Hair Cells by Forced Atoh1 Expression in the Adult Mammalian Cochlea

**DOI:** 10.1371/journal.pone.0046355

**Published:** 2012-09-27

**Authors:** Shi-Ming Yang, Wei Chen, Wei-Wei Guo, Shuping Jia, Jian-He Sun, Hui-Zhan Liu, Wie-Yen Young, David Z. Z. He

**Affiliations:** 1 Department of Otolaryngology—Head and Neck Surgery, Institute of Otolaryngology, Chinese PLA General Hospital, Beijing, China; 2 Department of Otolaryngology—Head and Neck Surgery, PLA Air Force General Hospital, Beijing, China; 3 Department of Biomedical Sciences, Creighton University School of Medicine, Omaha, Nebraska, United States of America; University of Southern California, United States of America

## Abstract

The hallmark of mechanosensory hair cells is the stereocilia, where mechanical stimuli are converted into electrical signals. These delicate stereocilia are susceptible to acoustic trauma and ototoxic drugs. While hair cells in lower vertebrates and the mammalian vestibular system can spontaneously regenerate lost stereocilia, mammalian cochlear hair cells no longer retain this capability. We explored the possibility of regenerating stereocilia in the noise-deafened guinea pig cochlea by cochlear inoculation of a viral vector carrying Atoh1, a gene critical for hair cell differentiation. Exposure to simulated gunfire resulted in a 60–70 dB hearing loss and extensive damage and loss of stereocilia bundles of both inner and outer hair cells along the entire cochlear length. However, most injured hair cells remained in the organ of Corti for up to 10 days after the trauma. A viral vector carrying an EGFP-labeled *Atoh1* gene was inoculated into the cochlea through the round window on the seventh day after noise exposure. Auditory brainstem response measured one month after inoculation showed that hearing thresholds were substantially improved. Scanning electron microscopy revealed that the damaged/lost stereocilia bundles were repaired or regenerated after Atoh1 treatment, suggesting that Atoh1 was able to induce repair/regeneration of the damaged or lost stereocilia. Therefore, our studies revealed a new role of Atoh1 as a gene critical for promoting repair/regeneration of stereocilia and maintaining injured hair cells in the adult mammal cochlea. Atoh1-based gene therapy, therefore, has the potential to treat noise-induced hearing loss if the treatment is carried out before hair cells die.

## Introduction

Noise-induced hearing loss (NIHL) is a major health problem. Acoustic trauma causes NIHL when permanent cochlear damage results from exposure to high-intensity sounds, such as explosions, gunfire, and firecrackers. NIHL is usually due to destruction of cochlear hair cells and/or damage to their hair bundles [1]. Cochlear hair cells transduce mechanical stimuli into electrical activity [2]. The hair bundle, a staircase array of stereocilia of different heights, is the site of mechanoelectrical transduction [3]. The delicate hair bundle is susceptile to both acoustic trauma and ototoxic drugs. While hair cells in lower vertebrates and mammalian vestibular systems can spontaneously regenerate lost stereocilia [4]–[6], mammalian cochlear hair cells no longer retain this capability [7]. The inability of stereocilia to self-repair can subsequently lead to hair cell death and permanent hearing loss. Injured neonatal gerbil hair cells can live for 10 to 12 days *in vitro* after their stereocilia are destroyed [7]. If adult cochlear hair cells can likewise survive for a number of days after loss of stereocilia due to exposure to impulsive noise or ototoxic drugs, this window of opportunity could be crucial for potentially rescuing and repairing hair cells using therapeutic genetic or chemical interventions.

The transcription factor *atonal* belongs to the family of basic helix-loop-helix (bHLH)-containing proteins and plays an essential role in the development of the *Drosophila* nervous system [8]. In mammals, Atoh1 (also known as math1) has been shown to be essential for neurogenesis in the central and peripheral nervous system [9]–[12], and for the formation of several non-neural cell types [13], [14]. In the auditory system, Atoh1 plays a critical role for hair cell differentiation during development [15]–[17]. Embryonic Atoh1-null mice fail to generate cochlear and vestibular hair cells, as indicated by the absence of stereocilia and other hair cell-specific markers [15]. Furthermore, overexpression (or misexpression) of Atoh1 in *in vitro*, *in vivo*, and *in utero* cochleae results in the production of ectopic hair cells derived from non-sensory supporting cells [18]–[21]. These studies suggest that Atoh1 is important for hair cell genesis or apical specialization (i.e., stereocilia development and formation). We questioned whether forced expression of Atoh1 in the noise-deafened cochlea can promote repair/regeneration of the stereocilia destroyed by impulsive noise.

## Materials and Methods

### 1. Animals

Care and use of the animals in this study was approved by the Institutional Animal Care and Use Committee of the Chinese PLA General Hospital. Healthy (250–300 g) adult albino guinea pigs of either sex were used for the experiments. Hearing in both ears was measured using auditory brainstem responses (ABR) before and seven days after noise exposure. Only those animals whose hearing thresholds were normal before exposure and significantly elevated (≥60 dB SPL at all frequencies tested) after noise exposure, with no obvious sign of middle ear infection and damage, were selected for further experiments.

### 2. Impulsive Noise Exposure

Animals were put in a specially designed cage with their heads constrained. The cage was housed inside a sound isolation room. Each animal was continuously exposed to 200 rounds of simulated gunfire inside the soundproof room. The sound source was placed 20 cm away from each ear. The peak sound pressure level measured near the ear canal was approximately 164 dB SPL (C-frequency weighting). The impulse had B-duration of 10 ms. The interval between each pulse was 10 seconds.

### 3. Viral Construct and Inoculation

Replication-deficient recombinant adenoviruses (Ad5) with deleted E1 and E3 regions were used to construct Ad.*Atoh1-GFP* and Ad.*GFP* using the Adeno-X expression system (K1650-1, Clontech). Dr. W. Q. Gao from Genentech, San Francisco, CA, kindly provided us with the *Atoh1* plasmids. The sequence and other information about the constructs have been published elsewhere [18], [20], [21]. Animals were anesthetized with xylazine and ketamine, and kept warm using a 37°C warm pad. Viral vectors were inoculated into the cochlea through the round window either seven days or one month after noise exposure. 5 µl of viral suspension with concentration of 1×10^10^ total particles of purified virus per milliliter was inoculated.

### 4. Immunocytochemistry and Scanning Electron Microscopy

Cochleae were perfused with 4% formaldehyde in phosphate buffered saline (PBS) and treated with 0.2% Triton X-100/PBS. Goat serum (10%) was used to block nonspecific binding. The tissue was then incubated with an anti-C-mPres antibody [22] and washed with PBS, followed by incubation with secondary antibodies. To visualize the stereocilia, F-actin was labeled with rhodamine-phalloidin, as described previously [7]. An anti-myosin 7a antibody was also used to label the hair cells as described elsewhere [21]. A sample was mounted on glass slides with antifade solution (Prolong Antifade Kit, Invitrogen, Carlsbad, CA) before imaging on an Olympus FluoView FV1000 Confocal Microscope (Olympus (China)., Ltd., Beijing).

For scanning electron microscopy (SEM), the cochleae were fixed with 2.5% glutaraldehyde in 0.1 M sodium cacodylate buffer (pH 7.4) containing 2 mM CaCl_2_, washed in PBS, then post-fixed for 15 minutes with 1% OsO_4_ in the same buffer and washed. The tissues were dehydrated in an ethanol series, critical point dried from CO_2_, and sputter-coated with gold. The tissue was then examined using a Hitachi S-3700N scanning electron microscope.

### 5. ABR and Cochlear Microphonic Measurements

Details of ABR and cochlear microphonic (CM) measurements are described as previously performed [22]. During ABR and CM measurements, guinea pigs were anaesthetized with xylazine and ketamine. For ABR, needle electrodes were inserted at the vertex and pinna. ABRs were evoked with clicks and/or 5-ms tone pips (0.5 ms rise–fall, at 30/sec) with frequencies of 4, 8, 16, and 20 kHz. The response was amplified, filtered, and averaged using the Intelligent Hearing System (Intelligent Hearing System Corp., Miami, FL). Sound level was raised in 20- and/or 5-dB steps. At each level, 1,024 responses were averaged. Hearing threshold was determined by visual inspection. For CM measurement, a silver wire electrode was placed near the round window membrane. Responses to a 4-kHz tone burst were amplified, filtered, and averaged using the Intelligent Hearing System.

## Results

**Figure 1:**
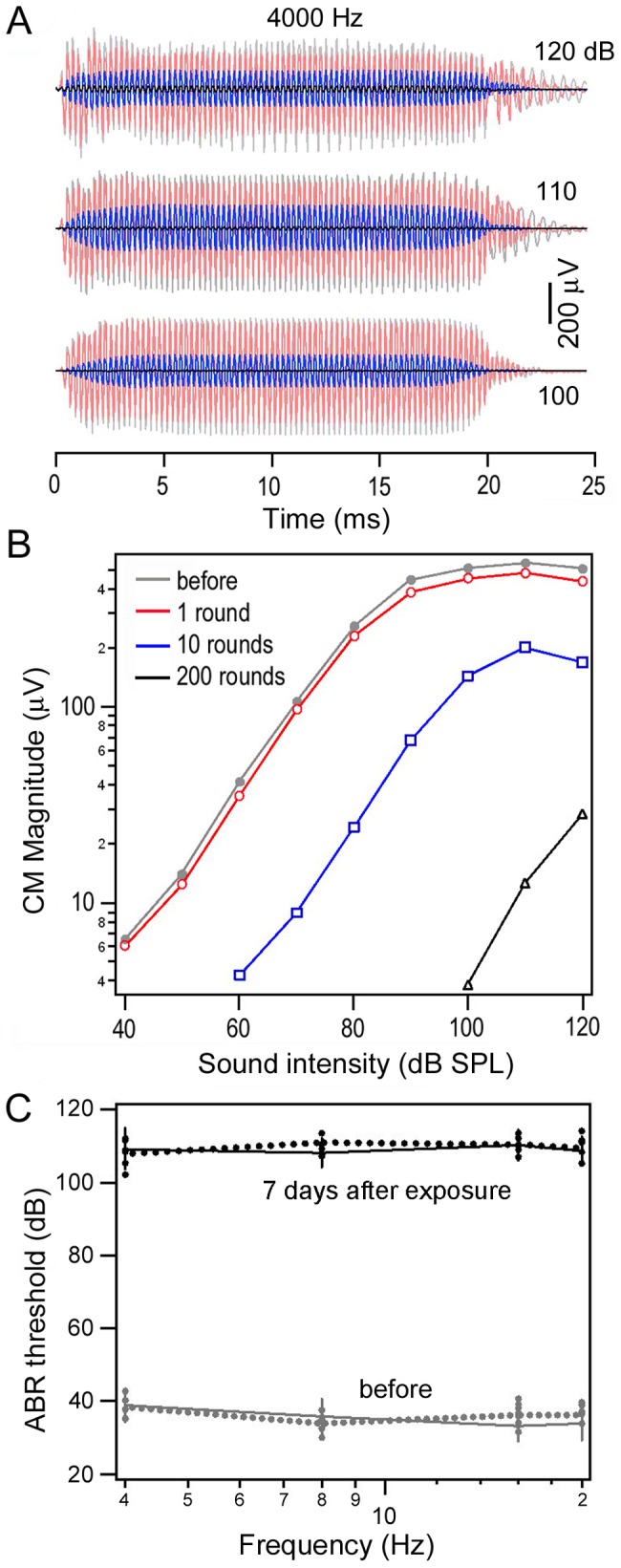
Hearing loss after exposure to simulated gunfire. *A:* Three examples of CM responses obtained before, during, and immediately after noise exposure. *B:* CM magnitude as a function of sound level before, during, and after noise exposure. Note the magnitude is plotted on log scale. *C:* Tone pip-evoked ABR thresholds (mean ± SD, n = 19) for both ears before and seven days after noise exposure. No statistical difference (student's t-test) in thresholds at each frequency was found between left (dot lines) and right (solid lines) ears either before or after noise exposure (p>0.05).

Adult guinea pigs were exposed to 200 rounds of simulated gunfire. We measured the CM to monitor the damage to the hair cells in some animals. The CM, an electrical potential generated in the cochlear hair cells in response to acoustic stimulation, primarily reflects mechanotransduction in the stereocilia of outer hair cells [23]. The CM response before and after 1, 10, and 200 rounds of gunfire was recorded. As examples, Figure 1A shows the CM response waveforms at three high sound intensity levels before, during, and after noise exposure. As shown, the magnitude of CM responses at saturation levels was reduced by as much as 26% after exposure to just one round of gunfire. After exposures to 10 and 200 rounds of gunfire, the magnitude decreased by 66% and 97%, respectively. The magnitude-intensity (input-output) function of the CM (Fig. 1B) shows that there was at least a 60 dB elevation in sound pressure level for the same magnitude of the CM after noise exposure. The reduction represents a significant disruption of outer hair cell (OHC) mechanotransduction after noise exposure. We measured hearing thresholds (Fig. 1C) before and seven days after noise exposure, using sound-evoked ABRs [22]. ABRs, frequently used to monitor hearing loss, are electrical signals evoked from the brainstem during presentation of an acoustic signal. ABR thresholds were obtained from both ears of 19 animals at 4, 8, 16, and 20 Hz using tone pips. The means and standard deviations of the thresholds before and seven days after noise exposure are presented in Figure 1C. Consistent with CM measurements, hearing thresholds for both ears were elevated by more than 60 dB for all frequencies tested. Since the ABR thresholds were measured at seven days after noise exposure to minimize the effect of temporary threshold shift, this threshold elevation should represent the permanent hearing loss. Only those animals whose hearing thresholds were elevated by more than 60 dB were used for further experiments.

**Figure 2:**
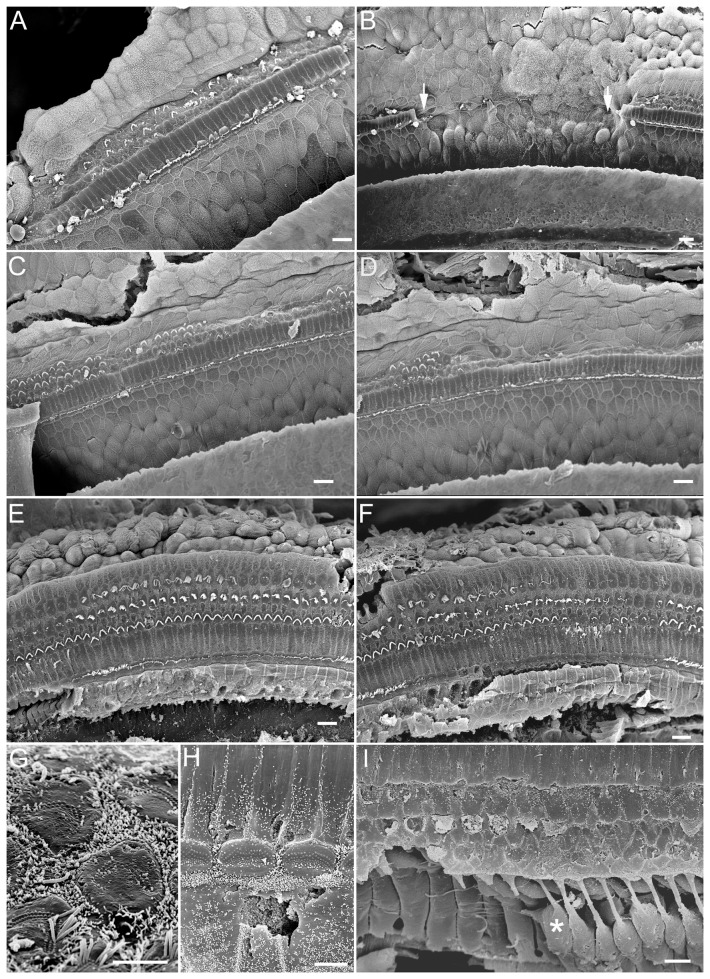
SEM images of the hair bundles seven days after exposure to impulsive noise. *A–F:* Images of hair bundle damage/loss obtained from representative locations in the basal (*A,B*), second (*C,D*), and third (*E,F*) turns. An area with hair cell death and scar formation is marked by arrows. *G,H:* High-magnification pictures of OHCs and IHCs with complete loss of stereocilia bundles. *I:* Image of complete loss of OHC stereocilia bundles in a second turn region. Note that the OHCs still remained in the organ of Corti despite loss of stereocilia bundles. A Deiters' cell is marked with an asterisk. Scale bars: 20 µm (*A–F*), 5 µm (*G,H*), and 10 µm (*I*).

We examined the morphological integrity of the stereocilia along the entire cochlear length at seven days after noise exposure using SEM. Figure 2 (A–F) shows examples obtained from three cochlear locations. Extensive stereocilia damage and loss of both inner and outer hair cells in the first, second, and third turns were observed. Sporadic hair cell death, as indicated by scar formation (marked by arrows in Fig. 2B), was also seen. Notably, injured hair cells remained in the organ of Corti despite severe bundle damage/loss. This is indicated by the presence of rootlets of the truncated stereocilia in the reticular lamina (Fig. 2G, H) and/or by the presence of OHC somas sandwiched between reticular lamina and the Deiters' cells (Fig. 2I).

**Figure 3:**
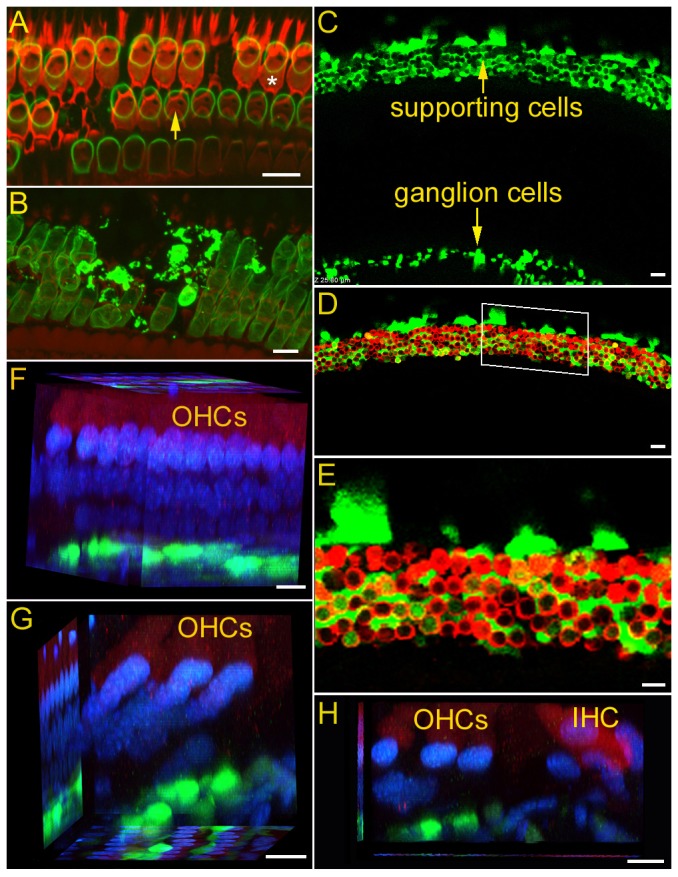
Confocal images of the stereocilia and prestin immunoactivity, and the pattern of Atoh1-EGFP expression in the organ of Corti. *A:* Stereocilia bundles in one cochlear location. The hair bundle was labeled with rhodamine-phalloidin (in red). The “V”-shaped OHC bundles were missing in the outmost (third) rows of OHCs. One bundleless OHC is indicated by an asterisk. One “V”-shaped hair bundle is marked by a yellow arrow. Prestin immunoactivity was labeled in green. Scale bar: 10 µm. *B:* Z-axis stack image of prestin immunoactivity at another cochlear location. Scale bar: 10 µm. All the images in *A* and *B* were obtained from cochleae 10 days after noise exposure. *C:* Confocal image of Atoh1-expression in a cochlea. *D:* Composite images of hair cells (labeled with myo7a antibody, in red) and EGFP-positive cells (from the same location as shown in panel *C*). The images were acquired seven days after Ad.*Atoh1-EGFP* inoculation in the noise-damaged cochlea (second turn). A magnified image of the area marked by white lines is presented in panel *E*. Scale bar: 20 µm for *C* and *D*. *E:* High magnification image of Atoh1 and myo7a expression in the organ of Corti. Scale bar: 10 µm. *F:* Confocal image obtained from optical sectioning from a basal turn location at 3 days after Atoh1 treatment. Hair cells were labeled with myo7a (in red) and the nuclei were labeled with DAPI. Most preparations examined at 3 days after transfection showed weak or no expression of EGFP in the organ of Corti. For those that expressed EGFP, the expression was in the area of Deiters' cells. *G, H:* Confocal image using optical sectioning from a basal turn location at 7 and 14 days after Atoh1 treatment. Scale bars (*F*,*G, H*): 10 µm.

To examine the fate of hair cells after stereocilia damage/loss, prestin immunoreactivity combined with confocal microscopy was used. Prestin is the motor protein of OHCs [24] and is only expressed in the plasma membrane [25]. Therefore, prestin immunoreactivity can be used as an assay to probe the status of OHCs. Absence of staining or presence of membrane debris would suggest cell death. To simultaneously visualize the hair bundle, rhodamine-phalloidin was used to label F-actin-containing stereocilia [7]. Figure 3A shows a composite confocal image obtained from a cochlea 10 days after noise exposure. As shown, the “V”-shaped OHC bundles (in red) were missing in the outmost (third) row and in some second row OHCs. However, the bundleless hair cells remained in the organ of Corti, although some sporadic hair cell loss was also observed (evidenced by empty space between prestin-labeled OHCs). Figure 3B shows a 3-D prestin-immunolabeled image (stacked along the z-axis) from a different cochlear location. This, again, shows that most OHCs survived the acoustic trauma despite sporadic OHC death (e.g., membrane debris and missing OHCs in Fig. 3B). Consistent with a previous study using the cultured organ of Corti [7], we concluded that noise-damaged OHCs with bundle damage/loss could survive up to at least 10 days after exposure.

Since the bundleless hair cells could remain in the organ of Corti for at least 10 days, this window could be crucial for intervention to repair hair bundles and to prevent the eventual death of hair cells. We explored the possibility of rescuing and repairing the injuried hair cells by overexpressing Atoh1. A viral vector carrying an EGFP-labeled *Atoh1* gene was inoculated into the cochlea through the round window on the seventh day after noise exposure. We chose the left ear for viral inoculation and the right (contralateral) ear as the control in eight animals. In another six animals, viral vectors carrying only the EGFP gene were inoculated into the left cochlea (seven days after noise exposure) as an additional control. Expression of Atoh1 by the EGFP expression was examined by confocal microscopy seven days after inoculation. Figure 3C exhibits a survey confocal image obtained from the basal turn in a whole-mount preparation. As shown, intense EGFP-positive labeling was observed in the areas of the organ of Corti and spiral ganglion neurons. To determine where the forced expression of Atoh1 occured in the organ of Corti, we used a myo7a antibody to label hair cells. Figure 3D shows that EGFP and myo7a were expressed in the different cell types in the organ of Corti. High magnification images show that EGFP expression was not in the hair cells (Fig. 3E). To further confirm that forced Atoh1 expression was not in hair cells, we took a series of optical sections to reconstruct 3-D images of the organ of Corti at 7 days after transfection. Reconstruction from the obtained images confirmed that forced Atoh1 expression was not seen in the hair cells, but rather in the supporting cells (Fig. 3G). To determine whether forced Atoh1 expression ever occurred in the hair cells at different time points after transfection, we also examined EGFP expression at 3 (Fig. 3F) and 14 (Fig. 3H) days after Atoh1 inoculation. As shown, forced Atoh1 expression was confined to the supporting cells and was not detected in the hair cells.

**Figure 4:**
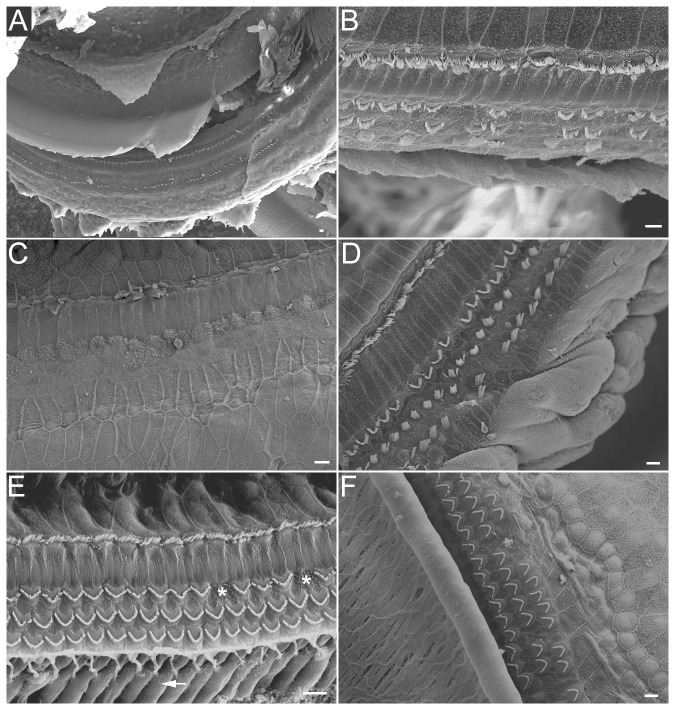
Representative SEM pictures obtained from different cochlear locations from Atoh1-treated and untreated (control) ears one month after viral inoculation. *A–C:* SEM images showing different extent and severity of damage from the control cochleae. *D–F:* SEM pictures of hair bundles from the Atoh1-treated ears. Missing hair bundles in panel *E* are marked by asterisks. The arrow indicates a Deiters' cell. Scale bars: 20 µm (*A*) and 10 µm (*B* to *F*).

The morphology of stereocilia at three cochlear locations was examined in the control (right) and Atoh1-treated (left) ears using SEM at one month after viral inoculation. In the control ears without Atoh1 inoculation after noise exposure, the reticular lamina was mostly flat with a few remaining stereocilia bundles (Fig. 4A–C). The remaining bundles had obvious signs of damage such as truncation, fusion, and folding. In contrast, most hair bundles in the Atoh1-treated ears appeared to have a normal appearence. Although hair cells with damaged or lost bundles were also sporadically observed along the entire length of the cochlea in the Atoh1-treated ears, most areas contained four rows of stereocilia (i.e., one row of IHCs and three rows of OHCs) on the reticular lamina (Fig. 4D–F). The Deiters' cells were coupled to the synaptic poles of OHCs (Fig. 4E). The presence of both OHCs and Deiters' cells suggests that the architectural relationship between OHCs and Deiters' cells was maintained in the “recovered” organ of Corti.

**Figure 5:**
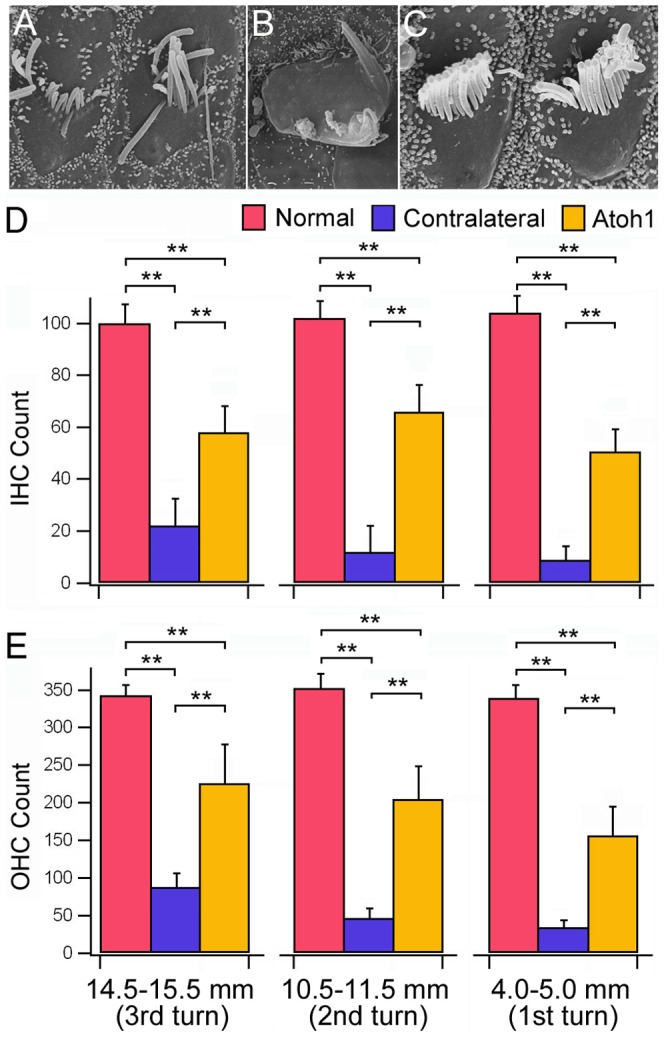
Stereocilia bundle counts in the normal (without noise exposure), Atoh1-treated and untreated cochleae. *A–B*: Examples of damaged bundles. *C*: Examples of normal-looking bundles. Scale bar represent 5 µm for all images in panels from *A* to *C*. *D, E*: Counts of IHC and OHC bundles in three different cochlear locations, each 1 mm in length. Three cochleae were used for each group (normal, Atoh1-treated, and untreated). Status of the bundles was determined by visual inspection of the bundles under scanning electron microscope. Hair bundles with no clear signs of trunction, fusion, or folding were included in the bundle count. Student's t-test was used for statistical analysis. Two asterisks represent statistical significance with a p-value less than 0.01.

To quantify bundle repair/regeneration after Atoh1 treatment, we counted the total number of bundles from three cochlear locations (each 1 mm in length) from three normal (i.e., without noise exposure) cochleae as reference. Cochleae from Atoh1-treated left ears, as well as from control (right) ears, at one month after inoculation were also examined. The three sites were approximately 14.5–15.5, 10.5–11.5, and 4.0–5.0 mm from the basal end of the basilar membrane. Although the characteristic frequencies of these locations were unknown due to the lack of a place-frequency map of the guinea pig cochlea [26], we estimated the characteristic frequency of these three locations based on recordings of inner hair cell (IHC) receptor potentials near these locations. Cheatham and colleague [27], [28] have determined that locations 17 (in the fourth turn), 15 (in the third turn), and 10 (in the second turn) mm from the basal end of the cochlea correspond to the characteristic frequencies of 270, 1000, and 4000 Hz, respectively. Based on this information, we estimated that the three locations we chose encompassed the areas that had the characteristic frequencies of roughly 1000, 4000, and 15,000 Hz, respectively. Three cochleae for each group of normal, Atoh1-treated, and untreated ears were used for bundle count. Status of the bundles was determined by visual inspection of the bundles under the scanning electron microscope. Hair bundles that had obvious signs of damage such as truncation (Fig. 5A), fusion (Fig. 5B), or folding were excluded in the bundle count. Although tip-links are known to be vulnerable to noise damage [29], [30], tip-link integrity was not considered in the bundle count.

Figure 5 (D and E) exhibits the means and standard deviations of the total numbers of bundles of IHCs and OHCs at three cochlear locations. As shown, noise exposure caused a significant reduction in the number of the hair bundles of both inner and outer hair cells in the untreated (right) ears (p<0.01 for both IHCs and OHCs at all three locations, student's t-test). After Atoh1 treatment, the numbers of bundles of both IHCs and OHCs were significantly greater than those of the untreated cochleae (p<0.01 for all cases). However, these numbers were still significantly less than in the normal cochlea, suggesting that a large number of damaged/lost bundles were not repaired. Transfection efficacy and hair cell death likely occurred before Atoh1 treatment were responsible for the disparity seen between the normal and Atoh1-treated ears.

**Figure 6:**
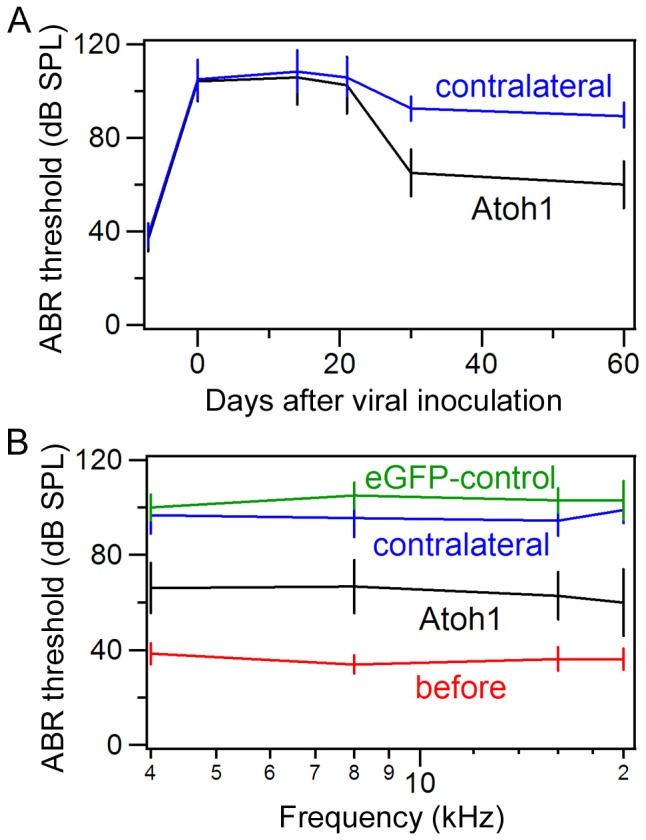
ABR thresholds measured from Atoh1-treated and control ears. *A:* ABR thresholds (means ± SD, n = 8) were measured at different days after the viral vector carrying the *Atoh1* gene was introduced. Contralateral ears were used as control. Click was used to evoke ABRs. *B:* ABR thresholds (means ± SD) of the Atoh1-treated ears (n = 8), contralateral ears (n = 8), and EGFP vector-treated ears (n = 6) at one month after cochlear inoculation. Tone pips with frequencies of 4, 8, 16, and 20 kHz were used to evoke ABRs.

**Figure 7:**
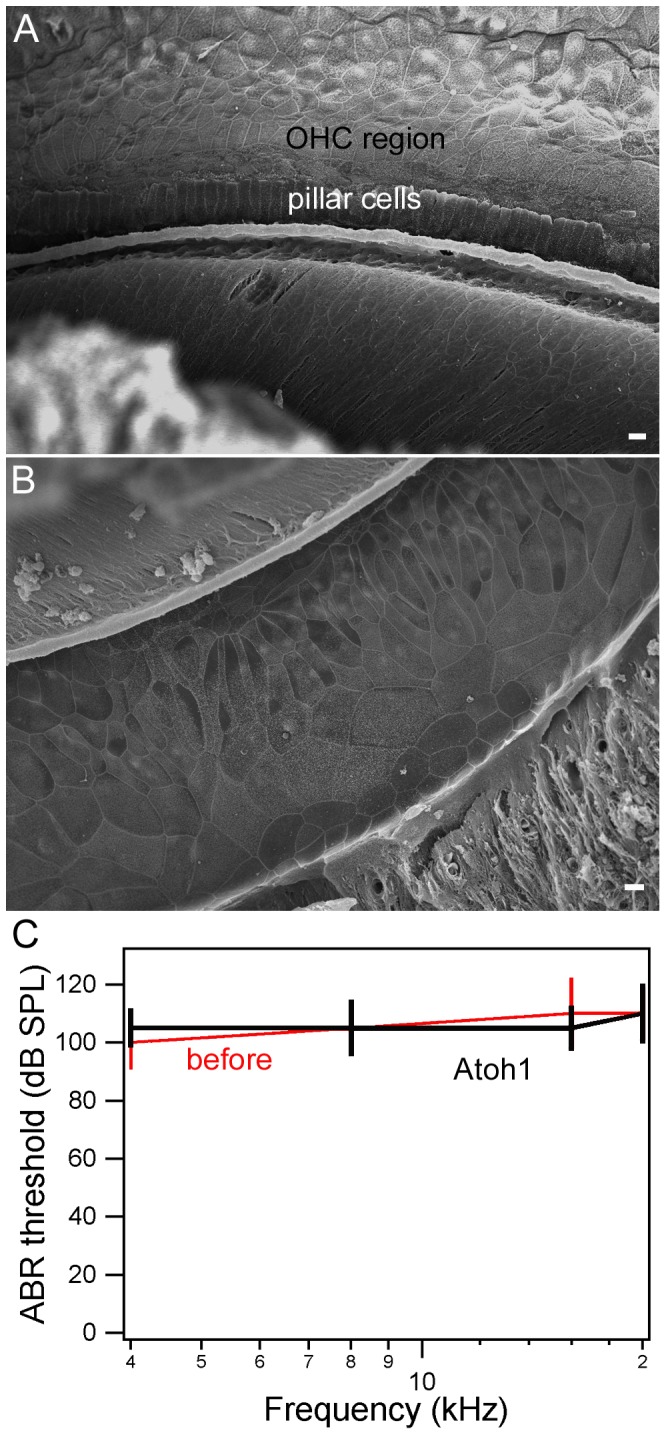
Representative SEM pictures and ABR thresholds obtained from cochleae treated with the Atoh1 gene one month after noise deafening. *A, B:* SEM images obtained from basal and middle turns. The reticular lamina was flat with no sign of stereocilia. Bars represent 10 µm. *C:* ABR thresholds (means ± SD, n = 4) measured from control and Atoh1-treated ears one month after the treatment.

We measured hearing thresholds from both (Atoh1-treated and contralateral) ears from eight animals using click-evoked ABRs at two weeks, three weeks, and one and two months after viral inoculation. As an additional control, hearing thresholds were also measured from noise-deafened animals with inoculation of the EGFP vector alone in the right ear. The results are presented in Figure 6A. Atoh1-treated ears showed an approximately 35–40 dB (SPL) threshold improvement (comparing to the contralateral ears) 30 days after Atoh1 treatment. We measured tone pip-evoked ABR thresholds at 4, 8, 16, and 20 kHz at one month after viral inoculation. Significant threshold improvement was seen at all freqeuncies tested (Fig. 6B). In contrast, thresholds in the contralateral ears without Atoh1 treatment were only improved by about 10–15 dB. Although thresholds of the contralateral ears also recoverd somewhat (Fig. 6A,B), this improvement was likely due to neural input from the cross (nerve) fibers originating from the Atoh1-treated ears [20] since the improvement disappeared when the middle ear strcutures in Atoh1-treated ears were intentionally damaged (in two ears, data not shown). ABR thresholds measured from noise-deafened ears with inoculation of the EGFP vector alone did not show any improvement one month after inoculation (Fig. 6B). To determine whether Atoh1 treatment was still effective after hair cell death, we introduced the *Atoh1* gene into the cochleae one month after noise exposure. None of the four animals showed any improvement in ABR thresholds examined at one month after the “delayed” treatment (Fig. 7). SEM showed that the hair cells were completely lost and the reticular lamina was flat with no signs of any normal-looking stereocilia bundles (Fig. 7). The absence of sensory epithelia and lack of improvement in hearing threshold after the delayed treatment suggest that the Atoh1-based gene therapy was effective only when injured hair cells were still present in the organ of Corti.

## Discussion

In this study, we show for the first time that introduction of the Atoh1 gene into a noise-damaged adult cochlea one week after noise exposure was able to substantially improve the hearing thresholds. Morphological examinations revealed that the numbers of stereocilia bundles of both IHCs and OHCs in the Atoh1-treated animals were significantly greater than those of the untreated cochleae. The re-emergence of stereocilia bundles after Atoh1 treatment suggests that Atoh1 is able to induce repair/regeneration of the damaged and/or lost stereocilia in the noise-deafened cochlea. We speculate that the new bundles originated from elongation of the truncated stereocilia, since the roots of truncated stereocilia were still present (Fig. 2G,H). It is also possible that regeneration originated from the microvilli. Gorelik et al. [31] show that microvilli may act as an elementary “building block” for the assembly of the specialized structures on the apical surface of cells. The mechanism(s) by which Atoh1 alone can promote repair/regeneration of the hair bundles with a functional mechanotransduction apparatus in both types of hair cells is unknown. It appears Atoh1 does not exert direct influence on hair cells, because forced Atoh1 expression was not detected in the hair cells (Fig. 3). However, supporting cells with forced Atoh1 expression may release diffusible factor(s) that may then influence adjacent hair cell repair.

It has been shown that the structure of the hair bundle is dynamically maintained by regulating the turnover of the actin filaments in a process described as treadmilling [32]. Recent evidence suggests that myosin-3a can facilitate hair bundle elongation by transporting espin-1 to the plus ends of actin filaments [33] and that the interaction of myosin-15a and whirlin is important for hair bundle morphogenesis and elongation [34]–[35]. Thus, it is possible that forced Atoh1 expression in the supporting cells may ultimately influence genes involved in hair bundle morphogenesis and elongation in the adjacent injured hair cells. Alternatively, the factor(s) released by supporting cells may stabilize the injured hair cells, allowing them to live long enough to repair themselves by upregulating genes involved in hair bundle elongation [33]–[35]. This notion that Atoh1 is important for maintaining hair cell viability in the adult cochlea is supported by a recent study [36]. We should point out that the molecular mechanism of how and why Atoh1 is critical for hair cell differentiation is not clear, despite the fact that its critical role in hair cell morphogenesis was discovered some 13 years ago [15]. Regardless of whatever the mechanisms may be, our study reveals a role of Atoh1 that has not been revealed before. This new role in maintaining injured hair cells and subsequently promoting repair of damaged stereocilia in the adult mammalian cochlea has the potential to treat hearing loss after exposure to impulsive noise if gene therapy can be applied shortly after acoustic trauma.

It is important to distinguish between hair bundle repair/regeneration and transdifferentiation of supporting cells into hair cells after forced Atoh1 expression. Izumikawa et al. have shown that Atoh1 is able to induce hair cell regeneration in cochleae deafened by ototoxic drugs [20]. According to their study, the newly generated hair cells are likely formed by phenotypic conversion of supporting cells that remained in the deafened cochlea. However, there are no follow-up studies to determine how long the hair cells converted from supporting cells can live without supporting cells. Hair cells and supporting cells in the organ of Corti depend on each other; malfunction or apoptosis of one population can trigger degeneration of the other. Follow-up studies to determine whether a critical period during which the Atoh1-based treatment is effective are also lacking.

Our proposed mechanism for repair/regeneration is different from that of Izumikawa et al. [20]. In our model, we demonstrated that it was the regeneration/repair of the stereocilia rather than transdifferention of supporting cells. The strongest evidence to support this notion is based on the fact that both hair cells and supporting cells were present and that the architectural relationship between OHCs and Deiters' cells was maintained in the “recovered” cochleae after the treatment (Fig. 4E). If Deiters' cells had been converted to hair cells without mitosis, we would not have seen both supporting cells and hair cells in the Atoh1-treated cochlea. Furthermore, it has long been known that hair cells and supporting cells depend on each other for survival in the organ of Corti. Hair cell death can rapidly trigger signaling pathways that lead to supporting cell death [37], [38]. Thus, it is inconceivable that either supporting cells or transdiffferentiated hair cells could survive alone in the adult mammalian cochlea. Besides, although supporting cells have the capability for phenotypic conversion to hair cells, such capability of the organ of Corti is only demonstrated in prenatal and neonatal preparations where hair cells and supporting cells are still under development [18], [21], [22]. It is not clear whether supporting cells in the adult mammalian organ of Corti still retain the potential to transdifferentiate, although interdental cells may be able to convert to ectopic hair cells after *Atoh1* gene transfer [19]. Finally, regeneration of stereocilia occurred only when Atoh1 inoculation was performed before hair cell death. Atoh1 treatment at one month after noise exposure could no longer rescue hair cells and restore hearing.

We should point out that there are substantial differences in how the *Atoh1* gene was introduced into the cochlea and how the animals were deafened between our experiments and those of Izumikawa et al. [20]. In our study, Atoh1 was inoculated through the round window and entered into the perilymph, which bathed both hair cells and Deiters' cells. In contrast, Izumikawa et al. inoculated the gene into the endolymph via the scala media. In our study the animals were deafened by impulsive noise, while Izumikawa et al. used ototoxic drugs to destroy hair cells. These differences may explain different underlying mechanisms for the two studies. However, it is known that individual susceptibility to ototoxic drugs varies significantly among animals (i.e., lethal dosage for one animal may be only sublethal to another) and that sublethal dosage of ototoxic drugs often only destroys stereocilia [5], [6]. Thus, we entertain the possibility that the hearing recovery observed by Izumikawa et al. could also be the result of hair bundle repair/regeneration after Atoh1 treatment.

We are fully aware that there are other mechanical and metabolic injuries (besides stereocilia damage) to hair cells, supporting cells, ganglion neurons, the stria vascularis, and Reissner's membrane after noise trauma [39]–[48]. The severity and extent of damage depends on the type (continuous vs. impulsive), level, duration, and interval of the noise [40]–[48]. In this study, possible damage to structures other than the stereocilia was not examined since our focus was the damage and repair in the hair bundles. However, we were aware that damage to other structures may cause subsequent hair cell death and hearing loss. We also note that the total number of bundles of inner and outer hair cells in the Atoh1-treated ears was significantly less than that of the intact cochlea (Fig. 5D and E). The reduction in the number of functional stereocilia bundles, together with possible damage to the Reissner's membrane and stria vascularis, may explain why the hearing threshold was not fully recovered with Atoh1 treatment. We measured ABR thresholds at 4, 8, 16, and 20 kHz, representing the locations that are most vulnerable to impulsive noise in the animal models [41], [46]–[48]. Impulsive noise has a relatively wide spectrum and does more damage in the higher frequency region than in the lower frequency region [41], [46]–[48]. The frequencies we have measured represent a large area in the basilar membrane, sufficient to demonstrate the damage and the subsequent recovery, and to rule out any site-specific variability.

Some evidence suggests hair cell apoptosis resulting from continuous and/or low-level noise is likely caused by metabolic dysfunction [49], while damage to the basilar membrane (and the organ of Corti) and Reissner's membrane by impulsive noise usually originates directly from mechanical trauma [41], [46]–[48]. Thus, Atoh1-based gene therapy may not be suitable for the treatment of all types of noise-induced hearing loss. However, if Atoh1 treatment can stabilize injured hair cells and directly or indirectly promote repair, such treatment may be effective for treating both noise- and drug-induced hearing loss. In the context of acoustic trauma, this could be especially important for the prevention/treatment of hearing impairment in the allied troops serving in Afghanistan, where the duration of their combat missions could be arranged based on a seven- to 10-day treatment window after exposure to impulsive weapon noise.
